# Evolution of Specialization of *Cassida rubiginosa* on *Cirsium arvense* (Compositae, Cardueae)

**DOI:** 10.3389/fpls.2016.01261

**Published:** 2016-08-23

**Authors:** Michael G. Cripps, Sarah D. Jackman, Cristina Roquet, Chikako van Koten, Michael Rostás, Graeme W. Bourdôt, Alfonso Susanna

**Affiliations:** ^1^AgResearch Ltd.Lincoln, New Zealand; ^2^Laboratoire d’Écologie Alpine, Centre National de la Recherche Scientifique, Université Grenoble AlpesGrenoble, France; ^3^Bio-Protection Research Centre, University of LincolnLincoln, New Zealand; ^4^Institut Botànic de Barcelona – Consejo Superior de Investigaciones Científicas – Institut de Cultura de BarcelonaBarcelona, Spain

**Keywords:** Cardueae, thistles, weeds, biological control, *Cassida rubiginosa*, *Cirsium arvense*, host specificity

## Abstract

The majority of herbivorous insects are specialized feeders restricted to a plant family, genus, or species. The evolution of specialized insect–plant interactions is generally considered to be a result of trade-offs in fitness between possible hosts. Through the course of natural selection, host plants that maximize insect fitness should result in optimal, specialized, insect–plant associations. However, the extent to which insects are tracking plant phylogeny or key plant traits that act as herbivore resistance or acceptance characters is uncertain. Thus, with regard to the evolution of host plant specialization, we tested if insect performance is explained by phylogenetic relatedness of potential host plants, or key plant traits that are not phylogenetically related. We tested the survival (naive first instar to adult) of the oligophagous leaf-feeding beetle, *Cassida rubiginosa*, on 16 selected representatives of the Cardueae tribe (thistles and knapweeds), including some of the worst weeds in temperate grasslands of the world in terms of the economic impacts caused by lost productivity. Leaf traits (specific leaf area, leaf pubescence, flavonoid concentration, carbon and nitrogen content) were measured as explanatory variables and tested in relation to survival of the beetle, and the phylogenetic signal of the traits were examined. The survival of *C. rubiginosa* decreased with increasing phylogenetic distance from the known primary host plant, *C. arvense*, suggesting that specialization is a conserved character, and that insect host range, to a large degree is constrained by evolutionary history. The only trait measured that clearly offered some explanatory value for the survival of *C. rubiginosa* was specific leaf area. This trait was not phylogenetically dependant, and when combined with phylogenetic distance from *C. arvense* gave the best model explaining *C. rubiginosa* survival. We conclude that the specialization of the beetle is explained by a combination of adaptation to an optimal host plant over evolutionary time, and key plant traits such as specific leaf area that can restrict or broaden host utilization within the Cardueae lineage. The phylogenetic pattern of *C. rubiginosa* fitness will aid in predicting the ability of this biocontrol agent to control multiple Cardueae weeds.

## Introduction

The majority of phytophagous insects are specialized feeders with host plant ranges restricted to a single plant family or lower taxonomic group (Strong et al., 1984; Bernays and Chapman, 1994). Since specialization is the predominant feeding strategy of phytophagous insects, it is often considered a mechanism for speciation and generation of biological diversity (Mitter et al., 1988; Janz et al., 2006), yet the ecological and evolutionary forces that promote specialization are still poorly understood (Forister et al., 2012). The evolution of specialization is generally framed in the theory of fitness trade-offs, whereby utilization of one host in a particular environment comes at the expense of utilizing an alternative host (Futuyma and Moreno, 1988; Joshi and Thompson, 1995; Roff and Fairbairn, 2007). Through the course of natural selection, host plants that maximize insect fitness should result in optimal, specialized, insect–plant associations. The ecological selection pressures promoting specializing in natural communities are multiple and varied, and include factors such as time to locate and develop on suitable hosts (Doak et al., 2006), competition for resources (Svanbäck and Bolnick, 2007), and enemy-free space (Bernays, 1989). While these contemporary ecological pressures can be important factors promoting specialization they are undoubtedly constrained to some degree by evolutionary histories that also play a role in shaping specialized insect–plant associations (Winkler and Mitter, 2008; Futuyma, 2010). Most insect–plant associations share long evolutionary histories that can be traced millions of years, often to the Paleogene, 30–60 mya (Farrell, 1998; Currano et al., 2008). These long evolutionary associations have allowed phytophagous insects to adapt to the chemical and physical defensive traits of particular plant lineages and may explain the commonly observed pattern of insect host ranges being phylogenetically conserved. However, even specialized insects have limits to the concentration of chemical defenses or magnitude of physical resistance traits that they can contend with (Ali and Agrawal, 2012). Therefore, even though closely related plants often share the same defensive traits, the traits themselves can vary quantitatively, which may be more important in determining insect performance than phylogenetic relatedness.

Within the host ranges of specialized phytophagous insects, performance hierarchies are common, but the plant traits and the phylogenetic relationships of the host plants that might explain the differences in insect performance arey seldom identified. The increasing availability of comprehensive resolved phylogenies is permitting more explicit tests of hypotheses concerning the evolution of specialization, and could permit better prediction of insect host ranges, and the degree of host plant specialization (Ødegaard et al., 2005). If specialized phytophagous insects exhibit a phylogenetically conserved pattern of performance across their potential host range it would suggest that insects are tracking overall plant trait similarity to an optimal host and indicate that host plant specialization is largely governed by evolutionary history. Alternatively, if phylogeny is not a good predictor of insect performance it would suggest that specialization, although broadly bound by evolutionary history, is labile within a plant lineage, and that insects track key plant traits that may exist through convergent or parallel evolution.

Previously, the fitness of the leaf-feeding beetle, *Cassida rubiginosa*, was tested in relation to plant defensive traits in a phylogenetically controlled experiment with three *Cirsium* species (Cripps et al., 2015). The beetle is classified as an oligophagous feeder restricted to plants in the tribe Cardueae (thistles and knapweeds), but has a well-known primary host plant, *Cirsium arvense* (Zwölfer and Eichhorn, 1966). The herbivore defensive traits of Cardueae species have not been well characterized, but some key traits that we identified as plausibly providing defense against specialized herbivores were leaf flavonoid concentration and leaf structural defenses, which were measured as specific leaf area, and the proportion of leaf pubescence. Flavonoids are known to act as feeding deterrents or stimulants in many insect–plant systems, depending on their concentration (Harborne and Grayer, 1993; Simmonds, 2001), and are the predominant secondary chemical group in the Cardueae (Wagner, 1977; Jordon-Thaden and Louda, 2003). Similarly, leaf pubescence and toughness are well documented as herbivore resistance traits (Hanley et al., 2007), and were identified as key defensive traits reducing *Cassida* fitness on congeners of *C. arvense* (Cripps et al., 2015). This raised the question of whether or not host plant utilization within the Cardueae was driven by key defensive traits (i.e., any given Cardueae species could be equally suitable, depending on key traits), or if there was a broad phylogenetic pattern explaining host plant specialization (i.e., adaptation to an optimal host over evolutionary time, independent of defensive traits). We hypothesized that both defensive leaf traits and phylogenetic relationship would affect the survival of the beetle. To determine the component effects of plant phylogeny and defensive traits on *Cassida* survival we measured the phylogenetic distance of each Cardueae test species from the primary host, *C. arvense*, and measured several leaf traits (specific leaf area, leaf pubescence, flavonoid concentration, carbon and nitrogen content) that might explain differences in insect survival.

## Materials and Methods

### Study System

The Cardueae is one of the largest tribes of the Compositae family comprised of approximately 2400 species in five subtribes (Susanna and Garcia-Jacas, 2007), and are considered a monophyletic group with a nearly completely resolved phylogeny (Susanna et al., 1995, 2006; Barres et al., 2013). The tribe originated during the mid-Eocene, and subtribal diversification events occurred throughout the Oligocene-Miocene period (Barres et al., 2013). The current native distribution is almost entirely in the northern hemisphere, and all of the Cardueae test species used in this study are native to Eurasia, and were introduced to New Zealand (NZ) either inadvertently, or deliberately as agricultural or ornamental plants (Webb et al., 1988). All of the inadvertently introduced Cardueae species in NZ are considered agricultural weeds that vary from minor to extreme economic importance (Cripps et al., 2013). The Cardueae plants selected for this study span three of the five subtribes and include representatives from widespread, species rich genera (e.g., *Cirsium* and *Centaurea*), and species poor genera with narrow geographic ranges (e.g., *Cynara*, *Ptilostemon*), and therefore provide a good representation of the tribe (Susanna and Garcia-Jacas, 2007; Vilatersana et al., 2010).

The tortoise beetle, *Cassida rubiginosa* Müller (Coleoptera: Chrysomelidae), is native to the Palearctic region where it is one of the most common insect herbivores on *C. arvense* (Zwölfer and Eichhorn, 1966). It was deliberately introduced to NZ in 2007 as part of the biological control program against *C. arvense* (Cripps et al., 2011b). Although the beetle is oligophagous, with many host plants in the Cardueae tribe, its release as a biological control agent was considered safe since there are no native Cardueae plants in NZ (Webb et al., 1988), and potential damage to economic plants (e.g., artichoke) was considered acceptable in cost-benefit analyses (Barratt et al., 2010). In its native range, at least 21 Cardueae species have been recorded as host plants, most belonging to the subtribe Carduinae (true thistles); however, the beetle shows a marked preference for *C. arvense*, which is considered its primary host (Zwölfer and Eichhorn, 1966). The beetle is univoltine and completes its entire life cycle (egg, 5 larval instars, pupa, and adult) on the foliage of host plants. Both adults and larvae are leaf feeders. Adults overwinter under leaf litter debris in hedge rows, or forest margins. Upon emergence in spring adults seek out host plants where they deposit their egg masses (oothecae), typically on the underside of leaves. Larvae are confined to the host plant where their eggs are laid, or adjacent overlapping shoots, but cannot move long distances along the ground to a new host plant (Tipping, 1993).

### Collection and Preparation of Test Plants

All of the test plants were grown from seed, except *Onopordum acanthium* and *Ptilostemon afer*, which were collected as rosettes from natural field sites in October 2013 and directly transplanted into 12-l pots. Seeds were either collected from the field (2009–2013) or purchased from a commercial supplier (King Seeds NZ Ltd.). Seeds of each species were sown on 21 August 2013. Seedlings were grown in a glasshouse at AgResearch until 17 October, when all plants were transplanted into 12-l plastic pots, and shifted outside to the experimental location in a fenced compound area on the campus of AgResearch, Lincoln (S 43°38′20.54″; E 172°28′28.2″). The plants were arranged in a randomized complete block design with five replicate blocks (80 plants total). The plants were grown in a standard potting mix (54% aged bark, 45% sand, 1% nutrients, by weight) containing added nutrients as Osmocote^®^ 17–11–10 (N–P–K), lime, superphosphate, sulfate of potash, and calcium nitrate. Watering of potted plants occurred four times daily (every 6 h) through an automatic irrigation system.

### Collection and Application of *Cassida*

Adult *Cassida* beetles were collected on 4 and 5 November 2013 from two field sites (Winton and Manapouri) in Southland NZ, where they had been released as biocontrol agents in 2007 and 2008, respectively. All adult beetles were collected from *C. arvense*. Adult beetles were kept in 2.2-l ventilated plastic boxes and fed *C. arvense* shoot clippings. The beetle colony was maintained in a laboratory at AgResearch Lincoln at a constant room temperature (*ca*. 20°C). Egg masses that were laid on the leaves of the clippings were removed and placed on moist filter paper in Petri dishes.

From 15 to 21 November, 12 naïve first instar larvae (hatched within 12 h) were placed on new fully expanded leaves of each plant in the experiment (total of 960 larvae). Larvae were applied one block at a time (1 or 2 days per block). A polyester mesh bag (50-cm × 125-cm) supported by wire struts was placed over each potted plant. At the time of beetle larvae placement, plants in the experiment were either large rosettes (biennial species), or bolting (annual species). On 27 January 2014 each plant was cut at the base and inspected in the laboratory for adult beetles. All surviving individuals were adults by this time (no other growth stages, i.e., larvae or pupae, were found), and the number of individuals per plant surviving to the adult stage was recorded. The mean temperature over the duration of the experiment (15 November to 27 January) was 16.3°C (range 4.4–34.8°C), based on hourly temperature readings recorded in the compound area from a Tinytag^®^ (Gemini Data Loggers Ltd) data logger.

### Specific Leaf Area, Leaf Pubescence, Flavonoids, Carbon, and Nitrogen Analyses

Three leaves from each plant in the experiment (representative of the size and age of leaves that the beetles were feeding on) were taken on 3 December. A 1.0-cm diameter cork-borer was used to cut a leaf disk from each of the three leaves, avoiding the midvein of each leaf. The three disks from each replicate plant were dried in an oven at 50°C for 48 h and the dry weights were recorded to the nearest 0.1 mg. The specific leaf area (SLA) was calculated as the ratio of the leaf area to dry weight (mm^2^/mg) of the three disks. The remainder of the leaves were also dried and then ground in a Retsch^®^ centrifugal mill through a 1.0-mm sieve. After milling, tomentous leaf pubescence (tightly adhering, matted leaf hairs) was separated from the dry leaf tissue material (re-milled two or three times as necessary to separate all leaf pubescence material) and weighed separately. The proportion of tomentous pubescence of the total leaf dry weight was recorded.

Total flavonoids were determined according to Zhu et al. (2010) but volumes were adapted for 96-well microplates. Beetle larvae were still feeding on the plants at the time of sample collection (3 December), and it is possible that this could have induced specific flavonoids, and thereby altered concentrations relative to plants in an undamaged state. However, since herbivore induction typically triggers species-specific, and specialized responses (e.g., single compounds) (Karban and Baldwin, 1997) the comparisons between plant species were considered to represent constitutive flavonoid concentrations, and any induction effects were assumed to be minor. Dried leaf powder (100 mg) was weighed into a 25-ml screw cap bottle and extracted with 7 ml ethanol (70% v/v) in a sonicator bath for 30 min. The extract was filtered (LBS 0001.090, Labserv, NZ) and an aliquot of 100 μl was transferred to a 2-ml microreaction tube. For the colorimetric reaction, 100 μl NaNO_2_ (5%, w/v) and 100 μl Al(NO_3_)_3_ (10% w/v) were added. After each step, the solution was vortex mixed and left to react for 6 min. Finally, 1 ml NaOH (4% w/v) and 600 μl aqua dest was added to the reaction tube, which was then vortexed, and left for 15 min. The absorbance of the extract (175 μl) was read at 500 nm against the uncoloured sample solution blank using a spectrophotometer (Multiskan GO, Thermo Scientific, USA). Rutin (Sigma–Aldrich, Australia) was used as a standard. Each sample was measured in triplicate. The total carbon and nitrogen concentrations (%) of the leaf samples were analyzed using an Elementar Vario-Max CN elemental analyser (Elementar Analysensysteme GmbH, Hanau, Germany).

### Phylogenetic Data and Analyses

**FIGURE 1:**
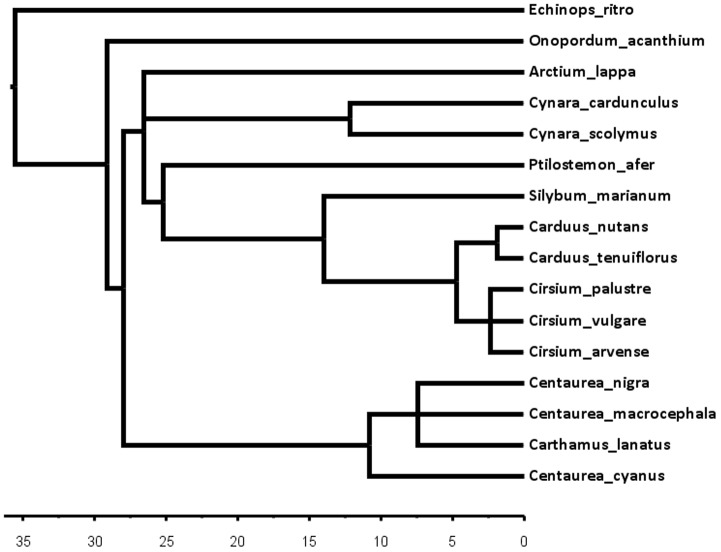
**Chronogram of the 16 Cardueae test species pruned from a comprehensive phylogeny of the tribe (Barres et al., 2013).** Branch lengths depict phylogenetic distances in millions of years (my).

The phylogeny of the 16 Cardueae test species was pruned from a comprehensive phylogeny of the Cardueae tribe based on nuclear ribosomal DNA and chloroplast DNA markers (Barres et al., 2013). Phylogenetic distance (in millions of years, my) was calculated from the total branch lengths separating each species from *C. arvense* using the function cophenetic.phylo (R package *ape*) (**Figure 1**).

Since survival was recorded as the number of *Cassida* adults from a group of 12 larvae that either survived or not, the percent survival values were analyzed using generalised linear models (GLMs), assuming binomial distributions through a logit link function (Faraway, 2005). The first seven GLM analyses examined if mean percent survival values were related to each of the seven explanatory variables alone (phylogenetic distance, SLA, flavonoids, percent pubescence, C, N, and C/N), i.e., the relationship of each individual variable to survival was examined independently from other variables. This was followed by further binomial GLM analyses that systematically examined if mean percent survival values were related to any combination of the seven explanatory variables using analysis of deviance. The sets of variables identified as statistically significant in the analysis of deviance were then compared based on their Akaike Information Criterion (AIC) values (Claeskens and Hjort, 2008).

The phylogenetic signal of all plant traits plus *Cassida* survival was estimated using Pagel’s lambda (λ) (Freckleton et al., 2002). Maximum likelihood estimates of the best λ value were compared to model estimates where λ was set at 0 (no phylogenetic signal, i.e., phylogenetic independence), or 1 (strong phylogenetic signal indicating the trait co-varies directly with shared evolutionary history).

We aimed to also test whether *Cassida* survival was related to plant traits while accounting for possible dependence among trait values due to shared evolutionary history. In recent years several methods have been developed to conduct phylogenetic regression analyses for continuous and binary data (for a review on methods for each type of data see Ives and Garland, 2014; and Symonds and Blomberg, 2014); however, to date there are no methods available for proportional data, such as survival percentages. Therefore, we applied arcsine square root transformations to survival proportions and performed phylogenetic generalised least squares (PGLS) regression (R packages *nlme*, Pinheiro et al., 2013, and *ape*, Paradis et al., 2004). Moreover, a recent study confirms that linear models applied to transformed data provide robust statistical tests for significance over a wide range of conditions (Ives, 2015). PGLS identifies from the phylogeny the amount of expected correlation between species based on their shared evolutionary history, and weights for this in the generalized least squares regression calculation using Pagel’s λ (it should be noted that in PGLS the assumptions regarding phylogenetic non-independence refer to the residual errors of the regression model, not the traits themselves). The phylogenetic structure might affect the covariance in trait values across taxa in different ways. Therefore, we compared two types of trait evolution model for each trait: Brownian motion models (BM), in which trait covariance between any pair of taxa decreases linearly with time since their divergence (three models diverging in lambda value were compared: fixed to 1, estimated from data, and fixed to 0); and Ornstein–Uhlenbeck models (Martins and Hansen, 1997), where the expected covariance decreases exponentially, as governed by the parameter alpha (values ranging from 0.5 to 10 were tested). PGLS was performed on all traits versus survival, each trait was first tested independently and then for all trait combinations in relation to *Cassida* survival.

## Results

**FIGURE 2:**
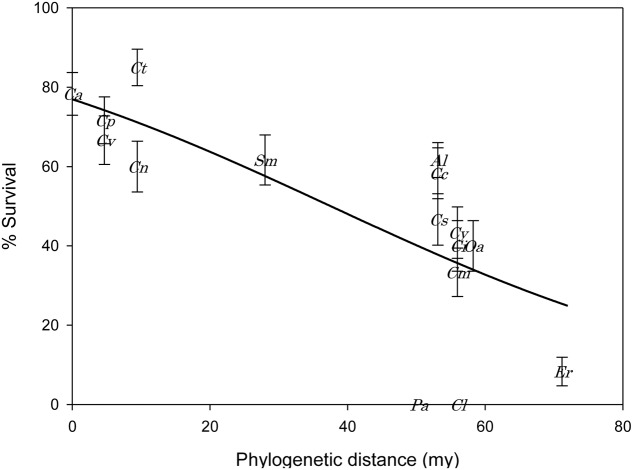
**Mean (±SE) percent survival of *Cassida rubiginosa* in relation to phylogenetic distance (million years, my) of each Cardueae test species from the primary host plant, *Cirsium arvense*.** The relationship between % survival and phylogenetic distance (PD) is given by the equation, % survival = 100/[1 + exp(-1.206 + 0.0321 × PD)]. Ct, *Carduus tenuiflorus*; Ca, *Cirsium arvense*; Cp = *Cirsium palustre*; Cv, *Cirsium vulgare*; Sm, *Silybum marianum*; Al, *Arctium lappa*; Cn, *Carduus nutans*; Cc, *Cynara cardunculus*; Cs, *Cynara scolymus*; Cy, *Centaurea cyanus*; Ci, *Centaurea nigra*; Oa, *Onoporodum acanthium*; Cm, *Centaurea macrocephala*; Er, *Echinops ritro*; Pa, *Ptilostemon afer*; Cl, *Carthamus lanatus*.

Mean *Cassida* survival (naïve first instar to adult) on the 16 Cardueae host plants ranged from 0 to 85% (**Figure 2**). The survival rates of *Cassida* on *Carduus tenuiforus*, *Cirsium palustre*, *Cirsium vulgare*, and *Arctium lappa* were statistically equivalent to *C. arvense*. Survival rates on the remaining 11 test plant species were significantly lower than on *C. arvense* (Duncan’s multiple comparison procedure, *P* < 0.05).

Of the traits measured, strong phylogenetic signal (Pagel’s λ) was detected for *Cassida* survival and leaf pubescence (**Table 1**). The strong phylogenetic signal of *Cassida* survival is a good indicator that phylogeny is a good predictor of *Cassida* performance, and that it can be used as a surrogate for plant traits influencing fitness of this species. Although leaf pubescence showed a phylogenetic signal (**Table 1**) it did not contribute significantly to *Cassida* survival.

**Table 1:** Phylogenetic signal (Pagel’s λ) for plant traits and survival of *Cassida rubiginosa*.

Trait	λ_estimated_	Significantly different from λ = 0	Significantly different from λ = 1
*Cassida* survival (%)	0.85	0.01	0.14
Leaf pubescence (%)	0.95	0.01	0.44
Flavonoid concentration (mg/g)	0	1	2.97 × 10^-4^
Specific leaf area (mm^2^/mg)	0	1	2.97 × 10^-4^
C (%)	0	1	1.36 × 10^-5^
N (%)	0	1	1.48 × 10^-3^
C:N ratio	0	1	6.40 × 10^-3^


*Values of λ close to 1 indicate a strong phylogenetic signal (resulting from shared evolutionary history), whereas values close to 0 indicate that phylogenetic signal is weak or null.*

**FIGURE 3:**
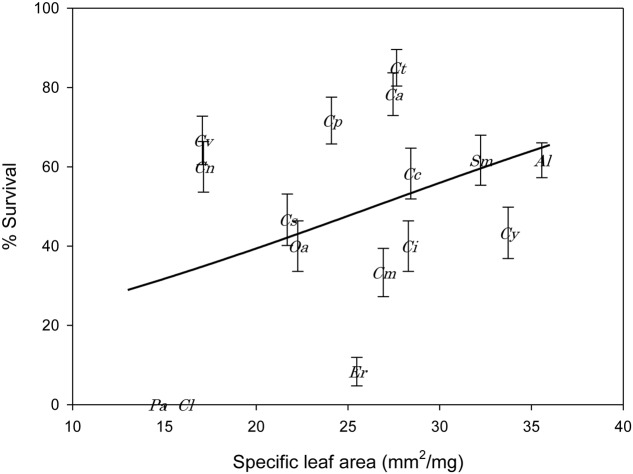
**Mean (±SE) percent survival of *Cassida rubiginosa* in relation to specific leaf area of the 16 Cardueae test species.** The relationship between % survival and specific leaf area (SLA) is given by the equation, % survival = 100/[1 + exp(1.770 – 0.0670 × SLA)]. Ct, *Carduus tenuiflorus*; Ca, *Cirsium arvense*; Cp, *Cirsium palustre*; Cv, *Cirsium vulgare*; Sm, *Silybum marianum*; Al, *Arctium lappa*; Cn, *Carduus nutans*; Cc, *Cynara cardunculus*; Cs, *Cynara scolymus*; Cy, *Centaurea cyanus*; Ci, *Centaurea nigra*; Oa, *Onoporodum acanthium*; Cm, *Centaurea macrocephala*; Er, *Echinops ritro*; Pa, *Ptilostemon afer*; Cl, *Carthamus lanatus*.

**FIGURE 4:**
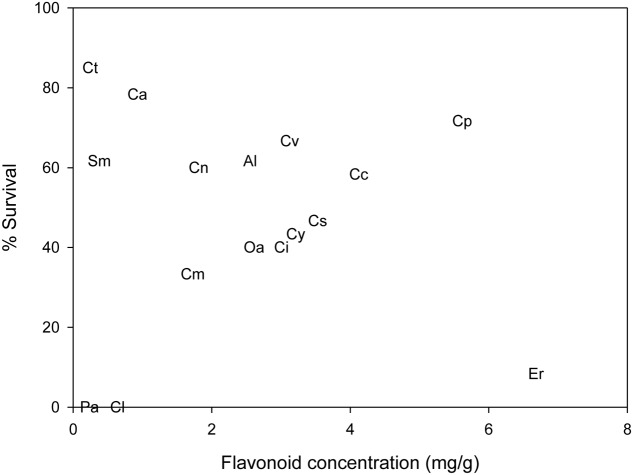
**Mean flavonoid concentration and percent survival of *Cassida rubiginosa* on 16 Cardueae test species.** The relationship between % survival and flavonoid concentration is not significant. Ct, *Carduus tenuiflorus*; Ca, *Cirsium arvense*; Cp, *Cirsium palustre*; Cv, *Cirsium vulgare*; Sm, *Silybum marianum*; Al, *Arctium lappa*; Cn, *Carduus nutans*; Cc, *Cynara cardunculus*; Cs, *Cynara scolymus*; Cy, *Centaurea cyanus*; Ci, *Centaurea nigra*; Oa, *Onoporodum acanthium*; Cm, *Centaurea macrocephala*; Er, *Echinops ritro*; Pa, *Ptilostemon afer*; Cl, *Carthamus lanatus*.

Based on the AIC values of significant GLMs, the most appropriate single-variable model predicting *Cassida* survival was phylogenetic distance, followed by SLA (**Table 2**). Survival of *Cassida* decreased with increasing phylogenetic distance from the primary host, *C. arvense* (**Figure 2**; **Table 2**), and this relationship remained significant after controlling for phylogenetic non-independence in the residuals (PGLS), indicating that phylogenetic distance itself (i.e., total branch length separation from *C. arvense*) is a good proxy for survival rates. Survival of *Cassida* increased with increasing SLA (**Figure 3**; **Table 2**), and this also remained significant after correcting for phylogenetic non-independence. From the single-variable GLMs, phylogenetic distance explained 58.4% of the variation in survival, and SLA explained 50.1% (i.e., the model correctly classified these percentages of individuals into survival/death groups). Similarly, based on the AIC values of significant multi-variable GLMs the most appropriate model was that of phylogenetic distance combined with SLA (**Table 2**). The GLM of phylogenetic distance + SLA explained 66.7% of the variation in *Cassida* survival. None of the other traits that were measured significantly explained survival of *Cassida* across the 16 test plants. In particular, total flavonoid concentrations did not explain *Cassida* survival (*P* = 0.413). The plant species with the highest survival rate (85%, *C. tenuiflorus*), and the species with the lowest survival rates (0%, *P. afer* and *C. lanatus*), had similarly low flavonoid concentrations (mean flavonoid concentrations for *C. tenuifolorus* = 0.2 mg/gDW; *P. afer* = 0.2 mg/gDW; and *C. lanatus* = 0.6 mg/gDW) (**Figure 4**).

**Table 2:** Plant traits predicting survival of *Cassida rubiginosa* resulting from the binomial generalised linear model (GLM) analyses.

GLM	Relationship to survival (%)	Significance	AIC
Phylogenetic distance (PD)	100/[1 + exp (-1.206 + 0.0321 × PD)]	<0.001	1158
Specific leaf area (SLA)	100/[1 + exp (1.770 – 0.0670 × SLA)]	<0.001	1216
PD + SLA	100/[1 + exp(0.973 + 0.0404 × PD – 0.0978 × SLA)]	<0.001	1105


*Lower Akaike Information Criterion (AIC) values indicate a closer fit to the data.*

In PGLS analyses, the most appropriate single-variable model predicting *Cassida* survival was SLA, followed by PD (phylogenetic distance), and C content (**Table 3**). According to the AIC values of significant multi-variable PGLS models, the most appropriate models are SLA + Percent pubescence, SLA + N content, SLA + flavonoids, and SLA + C:N ratio. All these models obtained very similar AIC values (**Table 3**). Only one of these four models (SLA + N) was significantly improved (according to likelihood ratio-test) by the addition of other variables: SLA + N + PD + C:N + Percent pubescence (**Table 3**). In this more complex model, survival decreases with phylogenetic distance, and increases with SLA, N content, C:N, and pubescence, although the final contributing variable (pubescence) was not statistically significant on its own.

**Table 3:** Plant traits predicting survival of *Cassida rubiginosa* resulting from phylogenetic least squares regression (PGLS) to account for possible dependence among trait values due to shared evolutionary history.

GLM	Relationship to survival (%)	λ	Significance	AIC
Phylogenetic distance (PD)	[sin (1.0836 – 0.0093xPD)]^2^	0	<0.0001	-1.13
Specific leaf area (SLA)	[sin (-0.1126 + 0.0272xSLA)]^2^	1	<0.0001	7.73
C content	[sin (2.0847- 0.0377xC)]^2^	1	<0.0001	12.75
SLA + % pubescence (PP)	[sin (-0.0806 + 0.0211xSLA + 0.0067xPP)]^2^	1	<0.0001	-21.64
SLA + flavonoids	[sin (-0.0974 + 0.0268xSLA – 0.0030x flavonoids)]^2^	1	<0.0001	-20.45
SLA + N	[sin (0.1215 + 0.0242xSLA – 0.0499xN)]^2^	1	<0.0001	-21.62
SLA + C:N	[sin (-0.2996 + 0.0245xSLA – 0.0194xC:N)]^2^	1	<0.0001	-21.35
SLA + N + PD	[sin (0.2729 + 0.0268xSLA + 0.0277xN – 0.0087xPD)]^2^	1	0.0001	-4.6
SLA + N + PD + C:N	[sin (-5.0978 + 0.0364xSLA + 0.7693xN – 0.0121xPD + 0.2252x CN)]^2^	1	0.0001	-15.61
SLA + N + PD + C:N + PP	[sin (-4.9092 + 0.0341xSLA + 0.7611xN -0.013xPD + 0.2157xC:N + 0.0036xPP)]^2^	1	0.0358	-18.02


*Lower Akaike Information Criterion (AIC) values indicate a closer fit to the data. Survival values were arc-sine square-root transformed. Phylogenetic signal associated with the regression was estimated with λ (values near 1 indicate strong phylogenetic signal of the residuals, values near 0 indicate null or weak phylogenetic signal).*

## Discussion

In accordance with our hypothesis, this study demonstrated that the survival of *Cassida* decreased with increasing phylogenetic distance from the primary host plant, *C. arvense*. This indicates that specialization is a conserved character, and that insect host range, to a large degree is constrained by evolutionary history. While the phylogenetically conserved pattern of insect performance has been long recognized (Wapshere, 1974), the novelty of the present work is that is uses a quantitative measure of evolutionary separation between hosts to predict insect herbivore performance across its host range (Pearse and Hipp, 2009; Rasmann and Agrawal, 2011). Phylogenetic distance is in essence a composite measure of trait similarity among the Cardueae test species, and is ultimately a good predictor of *Cassida* survival. However, the particular traits (chemical/physical) that account for the phylogenetic effect are still uncertain.

The only trait measured that clearly offered some explanatory value for the survival of *Cassida* across the 16 Cardueae test plants was SLA. This trait was independent of phylogeny, and when combined with phylogenetic distance from *C. arvense* was the best model explaining *Cassida* survival. This also conferred with our hypothesis that defensive traits, in addition to phylogenetic relationship, affect insect survival. Plant species with high SLA have many features conducive to herbivore feeding, such as lower dry matter, faster growth rate, and reduced leaf toughness (Salgado-Luarte and Gianoli, 2010), and this trait was shown to be an important factor explaining survival of *Cassida* on plants congeneric to *C. arvense* (Cripps et al., 2015). The only plant trait found to have a clear phylogenetic signal was leaf pubescence, but this trait was not related to *Cassida* survival. The fact that leaf pubescence showed a phylogenetic signal is likely an artifact of low sample size since this trait is common in plant species across the Cardueae subtribes (Susanna and Garcia-Jacas, 2007), and is therefore unlikely to be part of the underlying phylogenetic explanation for *Cassida* survival. Leaf pubescence is known to provide resistance against most types of insect herbivores, including both generalist and specialist feeders (Hanley et al., 2007), and previously trichome density was found to be an important factor determining survival rates of *Cassida* on plants congeneric to *C. arvense* (Cripps et al., 2015). In the present study, leaf pubescence was measured as a proportion of total leaf dry weight and specific pubescence structures that might differently affect *Cassida* survival were not distinguished. On *Cirsium* species, where the density of pubescence is important for *Cassida* survival, leaves are villose below and hirsute-hispidule above, and both types of hairs can create a barrier to the leaf surface. However, some other Cardueae species (e.g., *Cynara* and *Onopordum* spp.) have a relatively high proportion of leaf pubescence, but it is tightly adhering tomentous pubescence that does not appear to inhibit access to the leaf surface, and therefore does not affect *Cassida* survival.

Total flavonoid concentration did not show a phylogenetic signal, and did not explain *Cassida* survival. Interestingly, the host plant with the highest survival rate (85%, *C. tenuiflorus*) had a similarly low flavonoid concentration as the two non-host species (*P. afer* and *C. lanatus*). From other studies, there is mixed evidence with regard to the importance of flavonoids on insect performance, with inhibitory effects on some herbivore species, but no effects on others (Simmonds, 2001), regardless of being a generalist or specialist (Bi et al., 1997). While total flavonoids did not explain *Cassida* survival in this study it is likely that the specific flavonoid composition is important. In a related study, *Cassida* was shown to be tolerant to double the concentration of flavonoids on plants congeneric to *C. arvense*, which suggested that *Cassida* might be adapted to specific flavonoids that were common to plants closely related to *C. arvense* (Cripps et al., 2015). Although flavonoids are the predominant group of secondary chemicals in the Cardueae, other chemicals such as terpenoids and alkaloids have also been identified from Cardueae species, and could also play a role in resistance to herbivores (Wagner, 1977). Cardueae species share many secondary chemical compounds, but also have unique profiles, and it is possible that the underlying phylogenetic effect is explained by overall chemical similarity to the primary host (Jordon-Thaden and Louda, 2003). Much attention is often given to the role of defensive secondary chemistry; however, it is also possible that non-defensive chemicals might explain the phylogenetic effect on *Cassida* performance (Jermy, 1993). Insects often require sufficient stimuli for host recognition and sustained feeding that can be obtained through chemical attributes of the leaf surface, such as volatile organic compounds, epicuticular waxes, and primary metabolite exudates such as sugars and amino acids (Müller and Riederer, 2005).

Both phylogenetic relatedness and putative resistance traits independent of phylogeny (i.e., low SLA) contributed to the survival of *Cassida*, a conclusion also reached by similar studies (Becerra, 1997; Rasmann and Agrawal, 2011; Gilbert et al., 2015). These data indicate that specialization of *Cassida* on *C. arvense* has likely arisen through a combination of the beetle tracking phylogenetically conserved traits and responding to plant resistance characters that impose selection pressures for or against host utilization. This is in contrast to the conclusion reached by Slotta et al. (2012) who suggested that specialized herbivores on *Cirsium* and *Carduus* species do not follow a phylogenetic pattern, and that phylogeny does not predict host specificity. However, it should be noted that their conclusion was based only on literature records of known host associations and therefore the insect–plant associations for the bulk of species in their phylogeny are uncertain. Furthermore, the fact that a plant is recorded as a host does not mean it is an equivalent host in terms of insect fitness.

The potential to utilize other, more distantly related Cardueae plants, raises the question of what conditions might promote shifts in primary host use, and whether or not these could be sustained. The question is particularly pertinent in novel ranges such as NZ, where altered selection pressures are likely to exist, and may result in changes in the pattern of host plant use. Resistance characters might act to channel host plant selection and utilization without eliminating the innate ability of the insect to utilize other host plants, but whether or not resistance traits have evolved in response to herbivory is unclear. Traits such as low SLA (thick, tough leaves) and leaf pubescence are typically considered adaptations to xeric environments that primarily function to regulate temperature and conserve water (Johnson, 1975; Niinemets, 2001), and therefore these traits could be retained even in the absence of herbivory. However, if *Cassida* herbivory has fitness consequences for the plant, this could impose selection pressure for increased defensive traits (resistance and/or tolerance). This would depend on the degree of variation in defensive traits of thistles in NZ, but could result in reduced performance of *Cassida* over time. In the novel range of NZ, most Cardueae plants have experienced over a century of release from specialized insect herbivores (Cripps et al., 2011a), which may have allowed for evolution of reduced defenses (Bossdorf et al., 2005) that could, at least temporarily, enable increased fitness in *Cassida* on *C. arvense* and other Cardueae hosts.

Part of the reason for successful classical biological control is that insect biocontrol agents are released in novel, relatively benign environments, compared to their native ranges, and can achieve greater population numbers (Myers, 1987). The primary reason for this is release from the regulating influence of specialized predators and parasitoids. In NZ, *Cassida* experiences relaxed natural enemy pressure that allows population numbers of the beetle to increase far greater than observed in the native range (Cripps, 2013). Large numbers of the beetle might result in competition for the food resource and promote selection for alternative hosts, where competition is reduced, and survival rates are greater. Changes in the relative abundance of host plants due to successful biological control of one species, or land management changes that favor particular species, could also promote a shift in primary host plant utilization. Even with fitness trade-offs on alternative hosts (e.g., due to resistance traits), a more abundant resource might contribute more to beetle population numbers, and therefore selection could act to favor the more abundant plant (Futuyma, 2010). In fact, regional differences in Cardueae species abundance are thought to have led to biotype development of specialized capitulum-feeding insects, and was suggested as a mechanism for sympatric or parapatric speciation over evolutionary time (Zwölfer and Romstöck-Völkl, 1991). Given that other Cardueae plants are suitable hosts for *Cassida*, and four species other than the primary host were determined to be equivalent hosts for larval survival, there is potential for an altered pattern of host use in NZ (Van Klinken and Edwards, 2002). This will largely depend on the ecological selection pressures acting on host use, and how these are balanced against possible fitness consequences of using an alternative host. Determining the ecological selection pressures (e.g., enemy-free space) that influence the realized host range of *Cassida* in NZ will be the subject of future investigation, and will reveal the potential for contemporary evolution in novel environments, and also aid in predicting the success of an oligophagous biocontrol agent for controlling multiple thistle weeds.

## Author Contributions

The study was conceived by MC, MR, and GB. The experimental work was carried out by MC, SJ, and MR. Phylogenetic data was provided AS, and analyses were carried out by CR and CvK. MC wrote the manuscript with contributions from all authors.

## Conflict of Interest Statement

The authors declare that the research was conducted in the absence of any commercial or financial relationships that could be construed as a potential conflict of interest.
